# Depression before and during-COVID-19 by Gender in the Korean Population

**DOI:** 10.3390/ijerph19063477

**Published:** 2022-03-15

**Authors:** Won-Tae Cha, Hye-Jin Joo, Yu-Shin Park, Eun-Cheol Park, Soo-Young Kim

**Affiliations:** 1 Department of Public Health, Graduate School, Yonsei University, Seoul 03772, Korea; wcha@chahealthsystems.com (W.-T.C.); hjjoo22@yuhs.ac (H.-J.J.); dbtls0459@yuhs.ac (Y.-S.P.); ecpark@yuhs.ac (E.-C.P.); 2 Institute of Health Services Research, Yonsei University, Seoul 03772, Korea; 3 Chief Operating Officer (COO), CHA Health Systems, Inc., Los Angeles, CA 90010, USA; 4 Department of Preventive Medicine, Yonsei University College of Medicine, Seoul 03772, Korea

**Keywords:** COVID-19, South Korea, depression

## Abstract

This study explored the association between Coronavirus disease (COVID-19) and depression by comparing Patient Health Questionnaire-9 (PHQ-9) results pre-pandemic (2019) and after the start of the pandemic (2020). Data of 444,051 participants (200,206 male (45.1%); 243,845 female (54.9%)) were obtained from the Korean Community Health Survey conducted from 2019 to 2020. The independent variable of interest in this study was the year, divided into binary categories, 2019 and 2020. The dependent variable was depression, measured by the PHQ-9 scale. This dependent variable was also binary, dividing those who are considered depressed or not by a cut-off score of 10. A logistic regression model was employed to examine the association. Our results reveal that compared to participants in 2019, patients from the study sample of 2020 were marginally more likely to be depressed, especially female patients (male OR: 1.092, 95% CI [0.998 to 1.195], female OR: 1.066, 95% CI [1.002 to 1.134]). Moreover, using the participants from the year 2019 as a reference group, those who appeared anxious in response to the COVID-19-related questions in the survey showed more tendency to have a PHQ-9 score of 10 or more. Compared to participants from the 2019 group, those from 2020 more likely to be depressed were those with no-one to contact in case of emergency due to COVID-19 (male OR: 1.45, 95% CI [1.26 to 1.66], female OR: 1.46, 95% CI [1.33 to 1.60]), and individuals with concerns regarding economic loss (male OR: 1.18, 95% CI [1.07 to 1.30], female OR: 1.11, 95% CI [1.04 to 1.18]) and infection of a vulnerable family member at home due to COVID-19 (male OR: 1.16, 95% CI [1.05 to 1.28], female OR: 1.09, 95% CI [ 1.02 to 1.16]).

## 1. Introduction

With the outbreak of the novel coronavirus disease COVID-19 in 2019, countries globally were immediately and significantly affected [1]. We assumed that one of the effects COVID-19 has given to society would be an increase in mental health problems among populations [2,3,4]. Depression is one of the major diseases people suffer from. It is detrimental, and it usually persists for many years once initiated [5]. There are countless causes of depression, including genetic factors [6], individual stress [7], lack of social interaction [8], and anxiety [9]. Innate determinants for this mental health problem, all of the above were provided by the COVID-19 phenomenon, possibly causing people to experience depression. A few previous studies exist regarding depression and COVID-19 in various countries. In a study from Hong Kong, it was found that the more people were anxious about COVID-19, the more likely they were to be depressed [10], as did a similar study in Ireland [11].That latter study indicated that the anxiety and depressive mood people experience are common phenomena occurring from COVID-19.

Since the virus is highly contagious, quarantine protocols and gathering restrictions were imposed at a national level in China, where the massive outbreak began [12]. Even though these measures were required to prevent the spread of the virus, regulatory quarantine may have caused damage to the emotional health of people. Quarantine and stay-at-home policies were spread and implemented internationally [13,14,15]. When the Middle East respiratory syndrome coronavirus (MERS-nCoV) was prevalent in Korea in 2015, many people, including patients, uninfected family members, and healthcare workers, were mandatorily isolated. At that time, among the people in quarantine not diagnosed with the MERS-nCoV infection, 7.6% and 16.6% manifested anxiety and feelings of anger, respectively. Furthermore, months after the cessation of the quarantine, 3.0% and 6.4% of the former patients continued to present symptoms of anxiety and anger, respectively [16]. Therefore, individuals with long-lasting traumatic effects of quarantine, caused by forced social isolation protocols, require mental health support and, in some cases, counseling [17,18].

Many aspects of everyday life have also been affected by the pandemic. First, the global economy was severely damaged due to the spread of the virus. Consequently, many people became unemployed, and employers struggled to sustain their businesses [19]. Owing to the reduction in the work capacity of small establishments, business hours, and the number of customers, an economic recession was imminent, both in Korea and globally. In the US, the Automatic Data Processing, Inc. payroll data reported a 14% decrease in employment rates between 15 February and 18 April 2020 [20,21]. Similarly, based on data collected between 6 and 11 May 2020, employment rates declined by 19% in the UK [22]. This economic downturn relates to the degradation of people’s mental and emotional health [23,24,25].

Second, lifestyles substantially changed after the pandemic. Previous studies have reported that female were more mentally affected by the pandemic [26,27]; this worsened after the outbreak [28,29]. The lives of parents with school children were significantly impacted regardless of their employment status [30,31]. Schools were either closing or adopting an everyother-day strategy, and minimizing contact between students. This required parents, and especially mothers in Korean culture, to take care of children staying at home. Irrespective of whether mothers were physically close to their children, including working mothers, they were under a significant amount of stress due to the situation. As a result, the use of alcohol or cigarettes, which could act as a stress relief, may have increased [32,33] Nevertheless, these stress relief instruments work temporarily, ultimately strengthening the linkage between depression in individuals and the pandemic [34].

Since the negative outcomes of the pandemic include depression and low mood, it is crucial to investigate the deterioration in the mental health of individuals and pay more attention to preventing the exacerbation of depression due to the pandemic. In this study, we attempted to investigate the association between depression in individuals and the pandemic by comparing relevant variables for the years immediately before and after the outbreak of COVID-19.

## 2. Materials and Methods

### 2.1. Data

Data from the Korea Community Health Survey (KCHS), conducted from 2019 through 2020, were utilized for this study. Data was collected by a trained researcher visiting the households selected as a sample, conducting interviews using a laptop equipped with a survey program called Computer Assisted Personal Interview (CAPI). 

The KCHS is conducted and managed by the Korea Disease Control and Prevention Agency. This survey—which includes a large portion of the population and contains basic questions regarding sociodemographic and economic factors—has been conducted since 2008 to support future health-related policies by understanding of the current health status and aforementioned key characteristics of the population. 

### 2.2. Participants 

The total number of participants in the study was 444,051:200,206 male (45.1%) and 243,845 female (54.9%). Those under the age of 18 did not participate in this survey. Study participants resided in different parts of Korea and exhibited different socioeconomic characteristics. This study used a publicly available secondary dataset from KCHS. The KCHS received Korea Centers for Disease Control and Prevention (KCDC) IRB approval (2016-10-01-P-A) in 2016. From 2017, the ethics approval for the KCHS was waived by the KCDC IRB as it does not fall under human subject research based on the enforcement rule of the bioethics and safety act [35,36,37].

### 2.3. Variables 

The dependent variable was depression, measured by the Patient Health Questionnaire-9 (PHQ-9), a dependable tool for measuring depression [38]. The PHQ-9 contains nine questions, inquiring about the recent thoughts of individuals to assess depression. [39] Participants who scored 10 or above were considered depressed, and those who scored under 10 were considered not depressed [40]. The cut-off score for the PHQ-9 was suggested by the KCHS user guidelines [41,42].

The independent variable of interest in this study was the year, divided into binary categories, which are 2019 and 2020. The period of data collection was from August to October in both years. We were interested in the level of depression in these years particularly because the official outbreak of COVID-19 assigned by World Health Organization (WHO) occurred at a mid-point between s 2019 and 2020, in February 2020.

Covariates were controlled, such as sociodemographic (age, marital status, education level, number of generations living in the household) and socioeconomic factors (region of residence, occupational status, and household income). Controls for health behaviors (tobacco or alcohol use, perceived health conditions) that could function as confounders in investigating the association between the year and depression were also applied. The number of generations per household was divided into 1, 2, and 3—indicating grandchildren, parents, and grandparents living together. Occupational status was divided into three categories: employer or self-employed, employee, and unemployed. The unemployed group included students, homemakers, or those who were preparing for a job. The region of residence was divided into four categories, where the selected regions reflected those that were most affected by COVID-19 [43,44]: Seoul (the capital city), the Kyunggi area (the most populated), and the Daegu and Kyungbuk areas, which were affected by mass infection in the spring of 2019. Other areas were divided based on whether they were considered urban or rural. 

### 2.4. Statistical Analysis 

**Figure 1:**
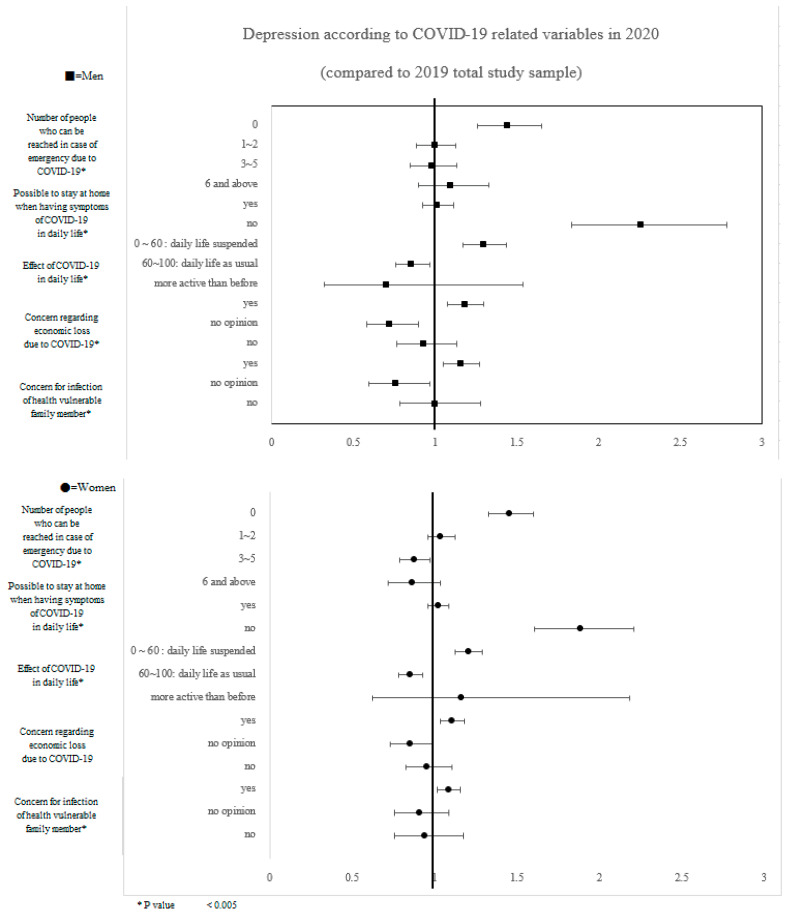
Depression according to COVID-19-related variables in 2020 (compared to 2019 total study sample).

To determine the association between the years before and after the outbreak of COVID-19 and depression, a binary independent variable of years 2019 and 2020, we performed logistic regression analysis. The results were reported using odd ratios (OR) and confidence intervals (CI). As shown in Figure 1, a 2019 study sample was employed as reference and the probability of depression was measured for each question related to COVID-19. The questions pertained to the following: the number of people, other than family members, who could be reached in case of an emergency due to COVID-19; the possibility of remaining at home if symptoms associated with COVID-19 were to develop; the effect of COVID-19 in everyday life on a scale of 0 to 100; fear of economic loss or infection of a vulnerable family member due to COVID-19. The data were analyzed and further stratified based on sex assigned at birth using SAS 9.4 (SAS Institute Inc.; Cary, NC, USA).

## 3. Results

Table 1 represents general characteristics of the study participants showing the frequency in each group. The depression rates were collected for two consecutive years, 2019 and 2020. The prevalence of depression was 2.1% in males and 3.8% in females. 

Table 2 presents the primary results of the logistic regression. For females, there was a minor increase in the odds ratio in 2020 compared to the data for 2019 (Male OR: 1.092, 95% CI [0.998–1.195]; Female OR: 1.066, 95% CI [1.002–1.134].) In the female group, those who lived with three generations in one household were more likely to be depressed, with statistically significant values (OR: 1.180, 95% CI [1.040–1.340]). In the male group, those who were unemployed were more likely to be depressed, with statistically significant values (OR: 1.504, 95% CI [1.311–1.726]). In categorizing the participants based on their region of residence, the Seoul and Kyunggi areas were found to have more depressed people compared to the rural areas of Korea (Male OR: 1.530, 95% CI [1.370–1.710]; Female OR: 1.329, 95% CI [1.230–1.435]).

Table 3 illustrates the results of the subgroup analysis stratified by independent variables. Using the 2019 sample as a reference, we analyzed each covariate. In the male group, regarding the inhabitation of the household by two generations, the 2020 participants were more likely to be depressed than the 2019 participants (OR: 1.16, 95% CI [1.01–1.33]). In comparison to 2019, in 2020 a higher number of generations living in one household corresponds with increased likelihood of depression for male and female participants. Regarding occupational status, compared to 2019, there was an increasing tendency of depression in all occupational categories, regardless of sex assigned at birth. Among different ages in the male group, participants in their 30s were significantly more likely to be depressed in 2020 than in 2019 (OR: 1.40, 95% CI [1.12–1.75]), and for the female group, participants in their 40s and 50s were more prone to be depressed in 2020 than in 2019 (40–49, OR: 1.23, CI: 1.03–1.45; 50–59, OR: 1.21, 95% CI [1.02–1.42]). As shown in Table 2, women who smoked cigarettes showed higher likelihood of depression in 2020 than in 2019 (OR: 1.35, 95% CI [1.11–1.65]), and individuals who perceived their condition of health as “bad” were more likely to be depressed in 2020 than in 2019 for males and females.

As shown in Figure 1, those who did not have anyone except close family members to contact in case of an emergency due to COVID-19 were more likely to be depressed in 2020 compared to 2019 (Male OR: 1.45, 95% CI [1.26–1.66]; Female OR: 1.46, 95% CI [1.33–1.60]). Those who could not remain at home when they developed symptoms of COVID-19 exhibited greater likelihood of depression compared with participants in 2019 (Male OR: 2.26, 95% CI [1.83–2.78]; Female OR: 1.88, 95% CI [1.61–2.21]). Those who responded that their daily lives were suspended due to COVID-19 were more likely to be depressed in 2020 than in 2019 (Male OR: 1.30, 95% CI [1.17–1.43]; Female OR: 1.21, 95% CI [1.13–1.29]). 

Compared to respondents in 2019, those in 2020 who expressed concerns regarding economic loss due to COVID-19 were more likely to feel depressed (Male OR: 1.18, 95% CI [1.07–1.30]; Female OR: 1.11, 95% CI [1.04–1.18]). Regarding concern for the infection of vulnerable family members at home, a similar tendency was exhibited; those who answered “yes” were more prone to being depressed in 2020 (Male OR: 1.16, 95% CI [1.05–1.28]; Female OR: 1.09, 95% CI [1.02–1.16]).

## 4. Discussion 

Our results reveal that compared to participants in 2019, the study sample of 2020 were marginally more likely to be depressed. Male respondents showed a higher odds ratio, indicating increased proneness to being depressed, but this was insignificant in statistics. The odds ratio for female respondents, on the other hand was statistically significant and they were more likely to be depressed during the pandemic than before. Moreover, using the participants from 2019 as a reference group, those who appeared anxious in response to the COVID-19-related questions in the survey showed more tendency to have a PHQ-9 score of 10 or more. Those who had no-one to contact in case of emergency due to COVID-19, people who were unable to remain at home when developing symptoms of COVID-19, those who reported that their daily life had been suspended due to COVID-19, and individuals with concerns due to COVID-19 about economic loss and infection of vulnerable family members at home were more likely to be depressed.

Previous studies have shown the relevant and absolute psychological impact of the outbreak of unfamiliar and powerful contagious diseases on individuals in general. It has been observed that severe anxiety and frustration related to infections hamper wellbeing and quality of life [45]. Thus, reinforcing social support can be an effective strategy for helping individuals cope with and adapt to the new environment [45,46]. Many studies have reported that those exposed to infections may have increased fears about their general health and worries about infecting others, particularly family members [47,48,49]. One study demonstrated that these people are more prone than others to expressing anxiety when they experience potentially infection-related symptoms. Even months later, they may believe that some symptoms are actively associated with the infection [16]. Moreover, other studies have shown that pregnant individuals and parents are most worried about becoming infected or transmitting the virus [50]. In Figure 1, we analyzed the likelihood of an individual from year 2020 being depressed, compared to the total 2019 study sample. Using the 2019 study sample as a reference, the questions that were asked only to the 2020 study sample were checked. Similar to the previous studies referred to above, those who answered they had no one to reach in case of emergency due to COVID-19, those who had no possibility of remaining at home when showing symptoms of COVID-19, those who thought their daily life was suspended due to the pandemic, and those concerned about infection of vulnerable family members at home were more likely to have depression, and the values were statistically significant. 

According to a previous study, female subjects were twice as likely to go through depression and other mental health problems compared to male counterparts [51]. Moreover, there is a study suggesting a biological mechanism that supports the claim; dysregulation of the hypothalamic–pituitary–adrenal axis and the sympathetic adrenal medulla has been identified in depression and anxiety disorders, and these disorders are triggered and exacerbated by stress [52]. In another study, from animal experiments, surprisingly, female rats were more resistant to stress inducers and did not have the impairment in memory that male rats had [53]. To accommodate these incompatible facts of prevalence and biological mechanism, one study suggests that male’s stress-causing neurobiological changes are adaptable, possibly preventing following progress of depression or anxiety problems [54]. In Korea, even though the social structure is rapidly changing, women still suffer from work–family conflict and are expected to be more involved than men in raising children [55]. We have divided gender into male and female in our study, because respective depressive behavior and prevalence differ, and our study results were in concordance, showing that females were more affected by depression after the change in the environment in 2020. There are few previous studies about depression during the pandemic. According to one study about quarantine, compared to those who did not go through quarantine, those who had experienced quarantine were more likely to have major depression [56]. This corresponds to our study, but the focus on quarantine differed from ours; social isolation and depression coming from the stay-at-home policy during the pandemic was the main point of our study. Another study into the association between perceived social support and depression during the pandemic had the same direction of conclusion as our study [57]; the more social support one gets from friends or family during the periods of need, the less likely one is to be depressed. We also concluded that these social distancing measures have made people more prone to depression.

This study was one of the first regarding depression before and during the pandemic to target the Korean population. Moreover, this study has strength in that we stratified participants by gender. As stated previously, depression is a gender-sensitive topic and therefore should be analyzed separately. Our study had a few limitations. First, it was a cross-sectional study design that did not target the same participants two years in a row. Nevertheless, since KCHS has a very large sample size, it is considered that those 200,000 people represent the entire Korean population. A longitudinal panel or cohort study should be conducted in the future. Second, secondary data collected by KCHS researchers were used for the study. Since the data were not collected by our researchers, some variables that needed to be considered for this study were not available in the dataset. For example, we would like to compare the comorbidities that individuals have and determine if they could have been one of the determinants or effect factors for people with depression. Lastly, the survey was conducted from August to October 2020, which was only half a year after the outbreak. There may be other challenges associated with the pandemic that may further impact the levels of depression and other negative health outcomes measured. Therefore, continuous research regarding this subject should be pursued. 

## 5. Conclusions

Our study demonstrated the association between the pandemic and depression, comparing the years immediately before and after the outbreak of COVID-19 using a PHQ-9 scale. Our results agree with those of many other studies and may be used as a policy-making guide for addressing the mental health deterioration of people resulting from the pandemic [56,57]. Since the outbreak, the Korean government has with great success focused on preventing the spread of the virus, which poses physical damage to people. During the early stages of COVID-19, introduction of drive-through screening centers, implementation of government policies to prevent the shortage of face masks, entry restrictions in public places, and stay-at-home restrictions were the methods the Korean government used to stop the growth spread of the novel virus [58,59]. Moreover, there have been few noticeable mass infections occurring in Korea, but local district government reaction to events in the cases that were reported was modeled internationally [60]. As government and society united and worked to hold back the virus from spreading, now it is time to pay attention to people’s emotional health affected by the pandemic.

## Figures and Tables

**Table 1:** General characteristics of the study subjects.

Variables	PHQ-9 Score ≥ 10 (Depression)											
	Male						Female					
	Total	Yes		No		*p*-Value	Total	Yes		No		*p*-Value
	N	N	%	N	%		N	N	%	N	%	
Total (N = 444,051)	200,206	4131	2.1	196,075	97.9		243,845	9356	3.8	234,489	96.2	
Year						0.0005						<0.0001
2019	99,396	2162	2.2	97,234	97.8		122,349	5060	4.1	117,289	95.9	
2020	100,810	1969	2.0	98,841	98.0		121,496	4296	3.5	117,200	96.5	
Generations residing in one household						<0.0001						<0.0001
One	96,149	2325	2.4	93,824	97.6		118,682	5110	4.3	113,572	95.7	
Two (parents and children)	91,743	1586	1.7	90,157	98.3		107,804	3607	3.3	104,197	96.7	
Three (grandparents and grandchildren)	12,314	220	1.8	12,094	98.2		17,359	639	3.7	16,720	96.3	
Occupational status						<0.0001						<0.0001
Employer or self employed	54,981	700	1.3	54,281	98.7		27,710	751	2.7	26,959	97.3	
Employee	87,789	1176	1.3	86,613	98.7		84,544	2234	2.6	82,310	97.4	
Unoccupied (students, housewives, etc.)	57,436	2255	3.9	55,181	96.1		131,591	6371	4.8	125,220	95.2	
Age						<0.0001						<0.0001
19–29	23,139	511	2.2	22,628	97.8		24,778	1137	4.6	23,641	95.4	
30–39	24,087	504	2.1	23,583	97.9		26,529	1008	3.8	25,521	96.2	
40–49	32,841	565	1.7	32,276	98.3		36,984	951	2.6	36,033	97.4	
50–59	39,285	619	1.6	38,666	98.4		46,656	1225	2.6	45,431	97.4	
60–69	39,470	671	1.7	38,799	98.3		47,819	1477	3.1	46,342	96.9	
≥70	41,384	1261	3.0	40,123	97.0		61,079	3558	5.8	57,521	94.2	
Marital status						<0.0001						<0.0001
Living with spouse	138,878	2174	1.6	136,704	98.4		148,261	4163	2.8	144,098	97.2	
Living without spouse	61,328	1957	3.2	59,371	96.8		95,584	5193	5.4	90,391	94.6	
Region						<0.0001						0.0001
Daegu, Kyungbuk	25,594	499	1.9	25,095	98.1		31,255	1175	3.8	30,080	96.2	
Seoul, Kyunggi	56,890	1309	2.3	55,581	97.7		68,148	2824	4.1	65,324	95.9	
Urban (Daejeon, Ulsan, Gwangju, Incheon, Busan)	32,568	684	2.1	31,884	97.9		40,308	1552	3.9	38,756	96.1	
Others	85,154	1639	1.9	83,515	98.1		104,134	3805	3.7	100,329	96.3	
Educational level						<0.0001						<0.0001
Middle school or less	51,295	1578	3.1	49,717	96.9		101,038	4928	4.9	96,110	95.1	
High school	63,488	1252	2.0	62,236	98.0		64,822	2213	3.4	62,609	96.6	
College or over	85,423	1301	1.5	84,122	98.5		77,985	2215	2.8	75,770	97.2	
Household income *						<0.0001						<0.0001
Below 2000	49,528	1980	4.0	47,548	96.0		78,034	4709	6.0	73,325	94.0	
Below 3600	37,820	685	1.8	37,135	98.2		42,460	1505	3.5	40,955	96.5	
Below 6000	53,701	812	1.5	52,889	98.5		57,585	1638	2.8	55,947	97.2	
6000 and above	59,157	654	1.1	58,503	98.9		65,766	1504	2.3	64,262	97.7	
Cigarette use (either conventional or electronic)						<0.0001						
Yes	67,520	1744	2.6	65,776	97.4		7116	857	12.0	6259	88.0	
No	132,686	2387	1.8	130,299	98.2		236,729	8499	3.6	228,230	96.4	
Current alcohol use						<0.0001						<0.0001
Frequently	64,194	1206	1.9	62,988	98.1		119,487	5185	4.3	114,302	95.7	
Occasionally	81,988	1314	1.6	80,674	98.4		102,832	3160	3.1	99,672	96.9	
None	54,024	1611	3.0	52,413	97.0		21,526	1011	4.7	20,515	95.3	
Perceived condition of health						<0.0001						<0.0001
Good	173,058	1871	1.1	171,187	98.9		194,166	3892	2.0	190,274	98.0	
Bad	27,148	2260	8.3	24,888	91.7		49,679	5464	11.0	44,215	89.0	

* unit: 10,000 won (₩).

**Table 2:** Results of factors associated with depression by year.

Variables	PHQ-9 Score ≥ 10 (Depression)									
	Male				*p*-Value	Female				*p*-Value
	OR	95% CI				OR	95% CI			
Year										
2019	1.000					1.000				
2020	1.092	0.998	-	1.195	0.0559	1.066	1.002	-	1.134	0.0418
Generations residing in one household										
One	1.000					1.000				
Two (parents and children)	0.949	0.848	-	1.062	0.6334	1.068	0.990	-	1.152	0.6705
Three (grandparents and grandchildren)	0.955	0.780	-	1.169	0.8348	1.180	1.040	-	1.340	0.0276
Occupational status										
Employer or self employed	1.000					1.000				
Employee	0.951	0.829	-	1.092	<0.0001	0.858	0.762	-	0.965	<0.0001
Unoccupied (students, housewives, etc.)	1.504	1.311	-	1.726	<0.0001	1.113	0.996	-	1.245	<0.0001
Age (years)										
19–29	2.335	1.873	-	2.909	<0.0001	2.801	2.417	-	3.246	<0.0001
30–39	2.947	2.408	-	3.606	<0.0001	2.665	2.302	-	3.084	<0.0001
40–49	1.910	1.583	-	2.304	0.0016	1.910	1.583	-	2.304	0.7575
50–59	1.327	1.124	-	1.567	0.0002	1.327	1.124	-	1.567	<0.0001
60–69	0.994	0.863	-	1.144	<0.0001	0.994	0.863	-	1.144	<0.0001
≥70	1.000					1.000				
Marital status										
Living with spouse	1.000					1.000				
Living without spouse	1.456	1.311	-	1.618	<0.0001	1.367	1.278	-	1.464	<0.0001
Region										
Daegu, Kyungbuk	0.981	0.841	-	1.143	0.0115	1.049	0.947	-	1.161	0.1222
Seoul, Kyunggi	1.530	1.370	-	1.710	<0.0001	1.329	1.230	-	1.435	<0.0001
Urban (Daejeon, Ulsan, Gwangju, Incheon, Busan)	1.051	0.926		1.192	0.1254	1.074	0.983		1.174	0.317
Others	1.000					1.000				
Educational level										
Middle school or less	1.484	1.263	-	1.745	<0.0001	1.307	1.157	-	1.476	0.0256
High school	1.242	1.104	-	1.398	0.6948	1.355	1.236	-	1.484	<0.0001
College or over	1.000					1.000				
Household income *										
Below 2000	2.262	1.944	-	2.632	<0.0001	2.114	1.901	-	2.351	<0.0001
Below 3600	1.494	1.276	-	1.749	0.5937	1.507	1.358	-	1.672	0.0384
Below 6000	1.333	1.165		1.526	0.0386	1.254	1.143	-	1.376	<0.0001
6000 and above	1.000					1.000				
Current cigarette use (either conventional or electronic)										
Yes	1.587	1.446	-	1.741	<0.0001	2.518	2.246	-	2.822	<0.0001
No	1.000					1.000				
Current alcohol Use										
Frequently	1.032	0.919	-	1.158	0.0120	1.555	1.400	-	1.727	<0.0001
Occasionally	0.826	0.740	-	0.921	<0.0001	1.097	1.024	-	1.175	0.000
None	1.000					1.000				
Perceived condition of health										
Good	1.000					1.000				
Bad	7.606	6.905	-	8.379	<0.0001	6.501	6.073	-	6.959	<0.0001

* unit: 10,000 won (₩).

**Table 3:** The results of subgroup analysis stratified by independent variables.

Variables	PHQ-9 Score of 10 or Above									
	Year									
	Male					Female				
	2020				2019	2020				2019
	OR	95% CI			OR	OR	95% CI			OR
Generations residing in one household										
One	1.00	0.88	-	1.13	1.00	1.06	0.98	-	1.16	1.00
Two (parents and children)	1.16	1.01	-	1.33	1.00	1.06	0.97	-	1.16	1.00
Three (grandparents and grandchildren)	1.21	0.82	-	1.77	1.00	1.11	0.88	-	1.39	1.00
Occupational status										
Employer or self employed	1.13	0.91	-	1.42	1.00	1.10	0.89	-	1.35	1.00
Employee	1.14	0.98	-	1.32	1.00	1.08	0.96	-	1.21	1.00
Unemployed (students, homemakers, etc.)	1.03	0.91	-	1.16	1.00	1.05	0.97	-	1.13	1.00
Age (years)										
19–29	1.08	(0.87	-	1.34)	1.00	1.01	(0.87	-	1.17)	1.00
30–39	1.40	(1.12	-	1.75)	1.00	1.07	(0.91	-	1.26)	1.00
40–49	0.94	(0.75	-	1.17)	1.00	1.23	(1.03	-	1.45)	1.00
50–59	1.12	0.90	-	1.41	1.00	1.21	(1.02	-	1.42)	1.00
60–69	1.07	(0.86	-	1.34)	1.00	0.97	(0.84	-	1.13)	1.00
≥70	0.93	(0.79	-	1.10)	1.00	0.98	(0.88	-	1.09)	1.00
Marital status										
Living with spouse	1.11	(0.98	-	1.25)	1.00	1.07	(0.97	-	1.17)	1.00
Living without spouse	1.06	(0.93	-	1.20)	1.00	1.07	(0.98	-	1.16)	1.00
Region										
Daegu, Kyungbuk	0.86	(0.66	-	1.11)	1.00	1.08	(0.91	-	1.28)	1.00
Seoul, Kyunggi	1.12	(0.97	-	1.29)	1.00	1.13	(1.02	-	1.24)	1.00
Urban (Daejeon, Ulsan, Gwangju, Incheon, Busan)	1.15	(0.95	-	1.41)	1.00	1.10	(0.96	-	1.27)	1.00
Others	1.10	(0.93	-	1.30)	1.00	0.94	(0.84	-	1.06)	1.00
Educational level										
Middle school or less	1.04	(0.88	-	1.22)	1.00	1.01	(0.93	-	1.10)	1.00
High school	1.07	(0.91	-	1.26)	1.00	1.07	(0.95	-	1.21)	1.00
College or over	1.14	(0.99	-	1.31)	1.00	1.11	(0.99	-	1.23)	1.00
Household income *										
Below 2000	1.00	(0.88	-	1.15)	1.00	1.02	(0.93	-	1.11)	1.00
Below 3600	1.14	(0.91	-	1.42)	1.00	1.03	(0.89	-	1.20)	1.00
Below 6000	1.15	(0.96	-	1.39)	1.00	1.19	(1.04	-	1.37)	1.00
6000 and above	1.12	(0.91	-	1.36)	1.00	1.04	(0.91	-	1.19)	1.00
Current cigarette use (either conventional or electronic)										
Yes	1.12	(0.98	-	1.27)	1.00	1.35	(1.11	-	1.65)	1.00
No	1.07	(0.95	-	1.21)	1.00	1.04	(0.98	-	1.11)	1.00
Current alcohol use										
Frequently	1.27	(1.08	-	1.48)	1.00	1.11	(0.93	-	1.31)	1.00
Occasionally	1.02	(0.87	-	1.18)	1.00	1.18	(1.07	-	1.31)	1.00
None	1.02	(0.88	-	1.18)	1.00	0.96	(0.88	-	1.04)	1.00
Perceived condition of health										
Good	1.05	(0.93	-	1.18)	1.00	1.04	(0.96	-	1.14)	1.00
Bad	1.15	(1.02	-	1.31)	1.00	1.10	(1.01	-	1.20)	1.00

* unit: 10,000 won (₩).

## Data Availability

The raw data was provided by the Korea Center for Disease Control and Prevention Agency (KDCA) from the Korea Community Health Survey (KCHS). The authors are completely accountable for the content of this manuscript; the article does not necessarily present the official position of the KDCA. URL: https://chs.kdca.go.kr/chs/rawDta/rawDtaProvdMain.do (accessed on 10 January 2022).
